# Functional Nanoparticles with Magnetic 3D Covalent Organic Framework for the Specific Recognition and Separation of Bovine Serum Albumin

**DOI:** 10.3390/nano12030411

**Published:** 2022-01-26

**Authors:** Lokesh Bettada, Hweiyan Tsai, C. Bor Fuh

**Affiliations:** 1 Department of Applied Chemistry, National Chi Nan University, Nantou 545, Taiwan; s105324905@mail1.ncnu.edu.tw; 2 Department of Medical Applied Chemistry, Chung Shan Medical University, Taichung 402, Taiwan; 3 Department of Medical Education, Chung Shan Medical University Hospital, Taichung 402, Taiwan

**Keywords:** magnetic 3D covalent organic frameworks, protein imprinted polymer, bovine serum albumin

## Abstract

Glutathione functionalized magnetic 3D covalent organic frameworks combined with molecularly imprinted polymer (magnetic 3D COF–GSH MIPs) were developed for the selective recognition and separation of bovine serum albumin (BSA). Ultrasonication was used to prepare magnetic 3D COFs with high porosity (~1 nm) and a large surface area (373 m^2^ g^−1^). The magnetic 3D COF–GSH MIP nanoparticles had an imprinting factor of 4.79, absorption capacity of 429 mg g^−1^, magnetic susceptibility of 32 emu g^−^^1^, and five adsorption–desorption cycles of stability. The proposed method has the advantages of a shorter equilibrium absorption time (1.5 h), higher magnetic susceptibility (32 emu g^−1^), and larger imprinting factor (4.79) compared with those reported from other studies. The magnetic 3D COF–GSH MIPs used with BSA had selectivity factors of 3.68, 2.76, and 3.30 for lysozyme, ovalbumin, and cytochrome C, respectively. The successful recognition and separation of BSA in a real sample analysis verified the capability of the magnetic 3D COF–GSH MIP nanoparticles.

## 1. Introduction

Covalent organic frameworks (COFs) are emerging porous crystalline polymers, constructed by combining light elements (e.g., B, C, Si, N, and O) through strong covalent bonds (e.g., B–O, C–N, C=N, and C=C–N), forming static ordered organic building blocks with pores of a precise size [1]. COF geometries are determined by the selection of different sizes, symmetries, linkers, and monomers. Studies have reported that COFs exhibit unique properties, such as ordered channels, a large specific surface area, highly tunable structure, and good thermal and chemical stability, leading to their outstanding performance in multiple fields, including energy conversion and storage [2], separation [3,4], gas adsorption [5], catalysis [6], and sensing [7]. Due to their unique photoelectric properties, COFs are demonstrating their potential as a platform for use in the biomedical field, such as for drug delivery, sterilization, bioimaging, and theranostics [8,9,10]. COFs can be classified into 2D and 3D COFs depending on the different ways the building blocks may be connected [11,12]. Compared with 3D COFs, 2D COFs are easier to synthesize because of their simple structure. They comprise ordered piled layers and are prepared using the ability of rigid organic units to generate individual nanosheets [12]. However, COFs have some drawbacks in terms of their applications, such as sedimentation and the time-consuming nature of the process, which can be overcome by the introduction of magnetic properties. A series of core-shell structured magnetic 2D COFs have therefore been synthesized and applied to various sample separations and analyses [13,14,15,16].

Compared with 2D COFs, 3D COFs have more sophisticated pore structures, such as interpenetrated channels and cages, which are beneficial during separation, catalysis, and guest incorporation processes, making them a superior platform. In addition, 3D COFs have more void framework structures creating large surface areas, low densities, and abundant accessible active sites, which are highly beneficial for practical applications [17].

In molecular imprinting technology, the polymerization reaction between monomer and cross-linker can form a polymer layer on the surface of template anchored nanoparticles. After eluting the template molecule, polymers form special binding sites for the target molecules in terms of size, structure, and functional groups [18,19]. Dummy template magnetic surface molecularly imprinted polymers (MIPs) that specifically recognized cyanidin-3-O-rutinoside have been designed [20]. Unfortunately, the finite surface area of the magnetic beads initially restricted the number of recognition sites, resulting in low adsorption capacity of the adsorbent. Therefore, MIPs based on COFs have been constructed to improve the capacity of MIPs [21]. Glutathione (GSH) is a tripeptide that possesses several types of functional groups, such as carboxyls, amines, and thiols. GSH enriches the ability of nanoparticles to sense their protein target and has been employed in the development of biorelated nanomaterials for various applications because of the multifunctional groups in its structure [22,23,24].

In this study, we add the magnetic 3D COFs that were covalently functionalized with GSH for the selective recognition of the targeted template molecules. Bovine serum albumin (BSA) was used as the template protein because it is the most common serum albumin, is easily obtained, and has a key physiological role in transporting various compounds. Tetraethyl orthosilicate (TEOS) was used as a cross-linker and 3-aminopropyl triethoxysilane (APTES) as a functional monomer to form a molecularly imprinted polymer layer because they are pH stable and reusable. The synthesized magnetic 3D COF–GSH MIP nanoparticles were characterized using a transmission electron microscope (TEM), Fourier transform infrared spectrometer (FTIR), thermogravimetric analyzer (TGA), and energy dispersive spectroscopy (EDS) elemental mapping. To evaluate the nanoparticles’ selectivity, the target protein was compared with three proteins (ovalbumin (OVA, MW 45.0 kDa, pI = 4.7), lysozyme (Lyz, MW 14.4 kDa, pI = 11.2), and cytochrome C (Cyt C, MW 12 kDa, pI = 10.2)) with near-spherical shapes but differing molecular weights [25,26].

## 2. Experimental

### 2.1. Materials and Chemicals

GSH, diglycolic anhydride (DA), 1-butanol, APTES, and iron chloride hexahydrate (FeCl_3_.6H_2_O) were obtained from Acros (Morris Plains, NJ, USA). BSA, Lyz, OVA, CytC, TEOS, sodium dodecyl sulfate (SDS), 1-ethyl-3-(3-dimethylaminopropyl) carbodiimide hydrochloride (EDC), N-hydroxysuccinimide (NHS), polyethylene glycol 6000 (PEG), ethylene glycol, and phosphate buffer saline (PBS) were purchased from Sigma–Aldrich (St. Louis, MO, USA). 1,3,5-triformylphloroglucinol (Tp) was purchased from Tokyo Chemical Industry (Tokyo, Japan). Tetra (p-aminophenyl) methane (TAM), 1,4*-dioxane*, and acetic acid (HOAc) were purchased from J.T.Baker (Philipsburg, NJ, USA).

### 2.2. Instrumentation

A UV–Vis spectrometer (Agilent Technologies, Cary 8454, Santa Clara, CA, USA), FT-IR (Frontier PerkinElmer, Waltham, MA, USA), TEM (JEOL, JEM-1400, Tokyo, Japan), and TGA (Cahn VersaTherm-HS TGA, Thermo Fisher, Waltham, MA, USA) were used in a flux nitrogen atmosphere. A superconducting quantum interference magnetometer supplied by Quantum Design (SanDiego, CA, USA) was also employed. Brunauer–Emmett–Teller (BET) analyses were conducted using a Micrometrics TriStar II Plus system (Norcross, GA, USA), with nitrogen employed as the adsorbate at 77 K. X-ray diffraction patterns of powder samples were acquired using a Shimadzu XRD-7000 system (Kyoto, Japan).

### 2.3. Preparation of COOH-Tp

COOH-Tp (cTp) was synthesized as shown in Scheme S1. Briefly, Tp (0.1 mmol) and DA (1.2 mmol) were separately dispersed in 25 mL of anhydrous tetrahydrofuran under ultrasonication (125 W, 20 KHz, 20 min). The Tp dispersion was slowly added to the DA solution at room temperature (RT) in a N_2_ atmosphere. The mixture was refluxed at 60 °C for 24 h and then cooled to RT. Subsequently, 25 mL of water was added to the mixture to suspend the reaction. After evaporation of the solvent, the yellow residue that remained (110 mg, 68% yield) was washed with water and dried under a vacuum. The obtained product was recrystallized using hexane and chloroform to obtain cTp, which was used for the synthesis of magnetic 3D COFs without further purification.

### 2.4. Preparation of Fe_3_O_4_@cTp-TAM (Magnetic 3D COFs)

Fe_3_O_4_-NH_2_ was synthesized in accordance with the method detailed in our previous study [27], and magnetic 3D COFs were synthesized using the method in [28,29], with some modifications. Briefly, through 20 min of ultrasonication, Fe_3_O_4_-NH_2_ (30 mg) and cTp (4 mg, 0.02 mmol) were dispersed in a mixture of solvents (1,4-dioxane (5 mL) and 1-butanol (5 mL)), also containing HOAc (100 µL). Subsequently, TAM (7 mg, 0.018 mmol) was added, and the mixture was then sonicated for a further 20 min and stirred for 30 min. Finally, the product was magnetically separated and washed multiple times with dry acetone and dried at 60 °C for 6 h.

### 2.5. Preparation of Magnetic-3D COF–GSH

**Scheme 1:**
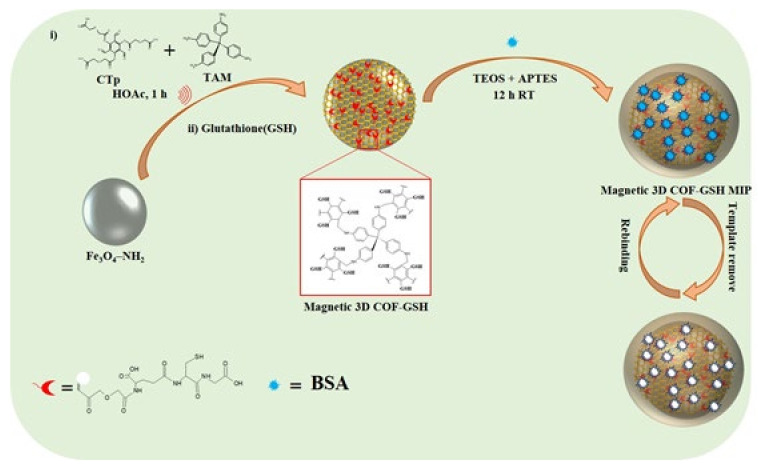
Preparation of magnetic 3D COF–GSH MIPs.

Magnetic-3D COFs were functionalized with GSH, as shown in Scheme 1 (ii). Briefly, magnetic-3D COFs (30 mg) and EDC (20 mg, 0.13 mmol) were dispersed in deionized water (30 mL pH-7.0), which was then sonicated for 30 min, after which they reacted at RT for 1 h. NHS (10 mg, 0.09 mmol) was added, the mixture was stirred for 1 h, and GSH (30 mg, 0.01 mmol) was then added and reacted for 6 h at RT. Finally, the product was obtained through magnetic separation and multiple deionized water washes, and the product was vacuum dried for 6 h.

### 2.6. Preparation of Magnetic 3D COF–GSH MIPs/Nonimprinted Polymers (NIPs)

As presented in (Scheme 1), magnetic 3D COF–GSH (3 mg) was dispersed in 5 mL of PBS (pH = 7.4), and 12 mg of BSA was added, with the resultant mixture then being stirred at RT for 30 min. TEOS (50 µL), APTES (20 µL), and NH_4_OH (100 µL) were then added, which was followed by stirring at RT for a further 12 h to achieve a polymerization reaction. The obtained magnetic 3D COF–GSH MIPs were washed twice with PBS to remove any unreacted BSA. The product was washed with 0.5% SDS and 0.1% HOAc (1:1) to remove template BSA molecules until absorption at 280 nm (BSA) had ceased for UV–Vis spectra of the supernatant solution. Magnetic 3D COF–GSH nonimprinted polymers (NIPs) were synthesized using the same procedure as that for the magnetic 3D COF–GSH MIPs, except no template (BSA) was added.

## 3. Binding Experiments

All the adsorption experiments were conducted at RT with a 500-rpm shaker. First, 1 mg of the magnetic 3D COF MIPs or NIPs was added to 1 mL of PBS solution (pH 6) containing BSA (0.1–1.0 mg L^−1^). After oscillation of the solution for 1.5 h, the particles were separated out using magnets, and the absorbance of BSA in the supernatant was determined using a UV–Vis spectrometer. The maximum absorption wavelength of the analytes BSA, Lyz, OVA, and Cyt C was 280, 280, 280, and 409 nm, respectively. The adsorption capacity (Q mg g^−1^) was calculated as follows [30].

Q = (C_o_ − C_e_) V/m (1)

where C_0_ (mg mL^−1^) and C_e_ (mg mL^−1^) are the initial and equilibrium concentration of the target molecules in solution, respectively. V (mL) is the volume of solution and m is the mass of the magnetic 3D COF–GSH MIPs or NIPs.

The imprinting effect of the magnetic 3D COF–GSH MIPs can be expressed through the imprinting factor (IF), calculated as follows.

IF = Q_MMIP_/Q_MNIP_ (2)

where Q_MMIP_ and Q_MNIP_ are the adsorption capacity of the magnetic 3D COF–GSH MIPs and NIPs to target molecules, respectively.

The separation effect of the magnetic 3D COF–GSH MIPs on BSA and the reference substances is represented by the separation factor (R), which was calculated as follows.

R = IF_tem_/IF_ref_ (3)

where IF_tem_ and IF_ref_ are the IF of the template protein and reference molecules, respectively.

The Langmuir (Equation (4)) and Freundlich (Equation (5)) absorption isotherm equations are as follows [31].

C_e_/Q_e_ = C_e_/Q_max_ + 1/(K_L_Q_max_) (4)

where Q_e_ (mg g^−1^) is the equilibrium adsorption capacity, Q_max_ (mg g^−1^) is the theoretical maximum adsorption capacity, and C_e_ (mg mL^−1^) is the concentration of the equilibrium solution.

logQ_e_ = 1/nlogC_e_ + logK_F_ (5)

where *n* is the adsorption intensity and K_F_ (mg g^−1^) is the adsorption capacity.

### Preliminary Application

In the preliminary application, 2 mg of the magnetic 3D COF–GSH MIP or NIP nanospheres were added to 1 mL of 0.1 mM PBS (pH 6) and spiked with 30 µL of BSA, Lyz, OVA, and Cyt C (1 mg mL^−1^). After a 2 h incubation, the magnetic 3D COF–GSH MIPs or NIPs were magnetically separated from the solution and washed with 0.5% SDS and 0.1% HOAc (1:1). UV–Vis spectra of the supernatant solutions were obtained. Gel-electrophoresis was also performed using SDS-PAGE (12% polyacrylamide separating gel and 5% polyacrylamide stacking gel); 30 μL of the product solution was removed for the SDS-PAGE electrophoresis.

In the real sample analysis, fetal bovine serum (FBS) was diluted 40-fold with a phosphate buffer (0.1 mM, pH 6). Approximately 5 mg of magnetic 3D COF–GSH MIPs or NIPs were added to 1 mL of FBS and reacted for 2 h. UV–Vis spectra of the supernatant solutions were obtained after washing with 0.5% SDS and 0.1% HOAc (1:1).

## 4. Results and Discussions

### 4.1. Preparation and Characterization of the Magnetic 3D COF–GSH MIPs

**Figure 1:**
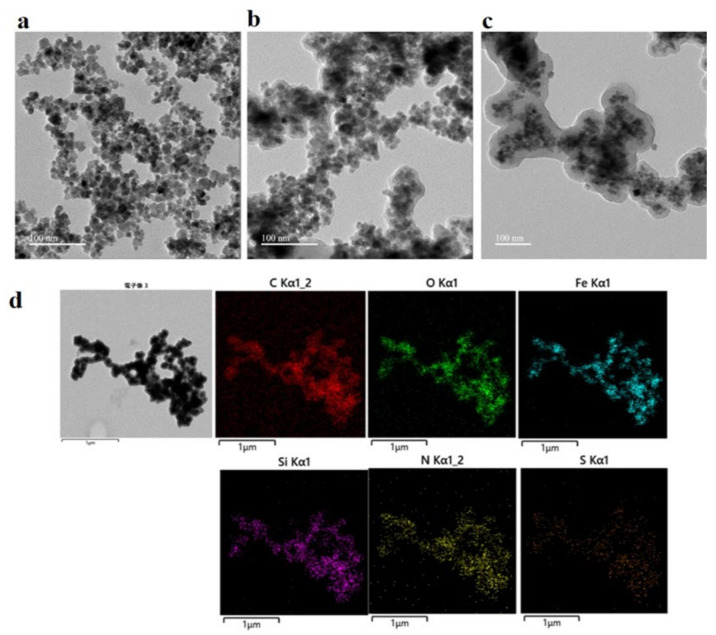
TEM images of (**a**) Fe_3_O_4_-NH_2_, (**b**) magnetic 3D COFs, (**c**) magnetic 3D COF–GSH MIPs, and (**d**) EDS elemental mapping of magnetic 3D COF–GSH MIPs.

Figure 1 presents TEM images of Fe_3_O_4_-NH_2_, the magnetic 3D COFs, and the magnetic 3D COF–GSH MIPs. The sizes of Fe_3_O_4_-NH_2_, magnetic 3D COFs, and magnetic 3D COF–GSH MIP nanoparticles were approximately 15 nm, 19 nm, and 35 nm, respectively. Figure 1d presents the EDS mapping of the magnetic 3D COF–GSH MIPs, showing that C, O, Fe, Si, N, and S were distributed throughout the material, indicating that the preparation was successful.

**Figure 2:**
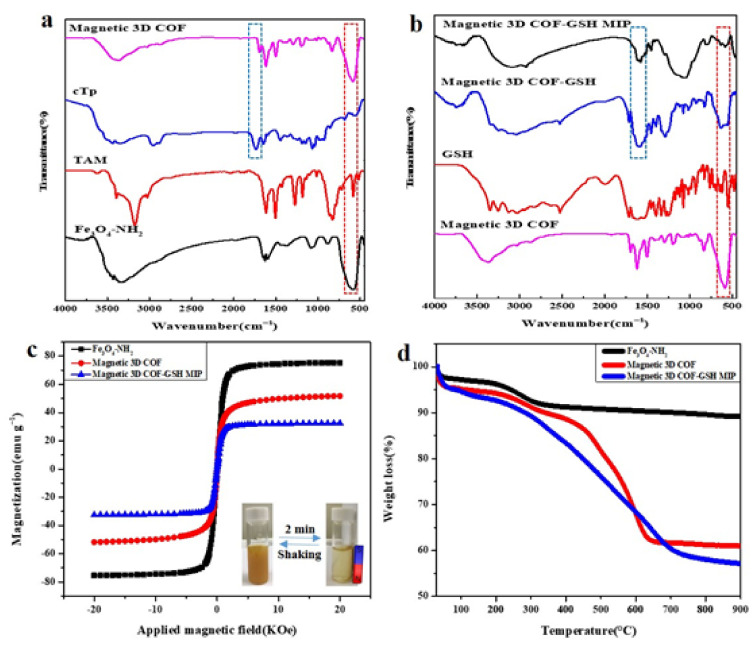
FT-IR spectra of (**a**) magnetic 3D COFs and precursors, (**b**) magnetic 3D COF–GSH MIPs and precursors, (**c**) magnetization curves of magnetic nanoparticles after each modification step, and (**d**) TGA analysis of magnetic nanoparticles.

The magnetic 3D COFs were prepared through imine-linked covalent bonding between cTp and TAM. Figure S1a,b depicts the FT-IR analysis, which verified the successful synthesis of cTp, with the characteristic peak at 1712 cm^−1^ representing the stretching vibration of –C=O from the carboxyl functional group. Figure 2a presents characteristic peaks at 1595, 1465, 1259, and 576 cm^−1^, which correspond to the –C=C, Ar (C=C), –C–N, and Fe-O in Fe_3_O_4_@cTp-TAM, respectively. Figure 2b shows peaks at 2920 and 2850 cm^−1^ (C–H stretching), 1657 cm^−1^ (C=O stretching of amide I), and 1540 cm^−1^ (C–S stretching), verifying the successful conjugation of GSH on the surface of the magnetic 3D COFs. Polymerization was confirmed by the characteristic peak at 1090 cm^−1^, corresponding to (Si–O–Si).

Figure 2c presents the magnetic hysteresis curve, demonstrating that the Fe_3_O_4_-NH_2_, magnetic 3D COFs, and magnetic 3D COF–GSH MIP materials possessed superparamagnetic characteristics and saturated magnetization values of 75, 51, and 32 emu g^−1^, respectively. Such high saturation magnetism is sufficient for a fast response to an external magnet for solid–liquid phase separation. As illustrated in the inset of Figure 2c, the magnetic 3D COF–GSH MIPs were well dispersed in water, forming a yellowish suspension, and were collected rapidly (2 min) with the help of an external magnet.

The thermal stability of the magnetic 3D COF–GSH MIPs was evaluated using a TGA, as depicted in Figure 2d. The Fe_3_O_4_-NH_2_ and magnetic 3D COFs had a weight loss of approximately 11.2% before 300 °C was reached, which was attributed to evaporation of absorbed water and residual solvents. The excellent thermal stability of the magnetic 3D COF–GSH MIPs was observed until the temperature reached 300 °C. According to the residual weight, the estimated weight loss for the magnetic 3D COFs and the final magnetic 3D COF–GSH MIPs was 39.8% and 44.8%, respectively.

The porous structure of the magnetic 3D COFs was confirmed by the N_2_ adsorption–desorption isotherms. The magnetic 3D COF materials exhibited a type IV isotherm (Figure S2) according to the IUPAC classification, indicating its mesoporous structure. The magnetic 3D COF materials exhibited a BET specific surface area of 373 m^2^ g^−1^ and pore volume of 0.186 cm^3^ g^−1^. A structure with pore diameters about 1.00, 1.35–1.85, and 2.16–3.18 nm was discovered (inset) for the magnetic 3D COFs. Massive carbonyl (–C=O) and amine (NH) groups of GSH can be functionalized with magnetic 3D COFs to enhance the adsorption capacity of BSA via H bonding. Figure S3 displays XRD of magnetic 3D COF nanoparticles. The crystalline morphology of the nanoparticles was also examined through XRD analysis. Figure S3 presents six diffraction peaks at 30.2° (220), 35.4° (311), 43.2° (400), 53.4° (422), 57.2° (511), and 62.4° (440) crystalline planes, which are characteristic peaks of Fe_3_O_4_. The similar XRD patterns of two nanoparticles indicate the successful preparation of magnetic 3D COFs without destruction of the framework’s integrity and crystallinity of Fe_3_O_4_. The diffraction peak of the magnetic 3D COFs’ spectrum appearing at around 17° could be attributed to the crystallinity of magnetic coated 3D COFs.

### 4.2. Optimization of the Adsorption Conditions

#### 4.2.1. Optimization of Monomer and Crosslinker on Adsorption

The amount of monomer (APTES) and crosslinker (TEOS) used is a key factor for achieving an optimal IF. In this study, we optimized and maintained the quantity of one chemical and employed three different quantities of the other, and vice versa (Table S1). The maximum amount of BSA extracted (Q) was achieved under the experimental conditions of 50 µL of TEOS and 20 µL of APTES. We used these conditions for subsequent experiments.

#### 4.2.2. Optimization of pH on Adsorption

**Figure 3:**
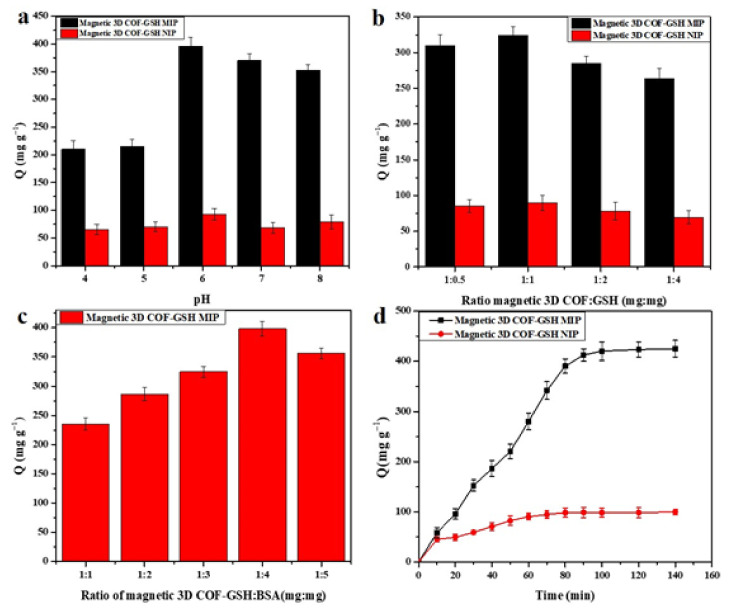
(**a**) Optimization of pH solution on adsorption, (**b**) optimization of the ratio between magnetic 3D COFs and GSH, (**c**) optimization of the ratio between magnetic 3D COF–GSH and BSA, and (**d**) adsorption kinetics of magnetic 3D COF–GSH MIPs and NIPs.

The effect of pH from 4 to 8 was investigated for the magnetic 3D COF–GSH MIP or NIP BSA imprinting. As indicated in Figure 3a, Q was very low at an acidic pH. This may have been because OH^−^ can nucleophilically attack the silica shell, creating surface defects in the BSA binding. The optimal Q and IFs were obtained at a pH of 6; this pH was therefore selected for further binding experiments.

#### 4.2.3. Optimization of GSH on Adsorption

In this process, we used GSH as a high-profile template protein capturing unit to achieve an improved Q for the targeted protein. Therefore, the amount of GSH used in the process of synthesizing the magnetic 3D COF–GSH MIPs or NIPs had to be optimized. We employed various GSH concentrations while maintaining a constant amount of M-3D COFs. Figure 3b illustrates that Q was at its maximum for a ratio of 1:1. We therefore used a ratio of 1:1 as the optimal condition for subsequent experiments.

#### 4.2.4. Optimization of BSA on Adsorption

The concentration of BSA as a template protein is a key factor in the process of synthesizing the magnetic 3D COF–GSH MIPs. Using the optimal amount of BSA in the synthesis process would result in a high separation factor. We employed five ratios in this process (1:1–1:5). As indicated in Figure 3c, the BSA adsorption capacity of the magnetic 3D COF–GSH MIPs was highest for a ratio of 1:4. This could have been because excess BSA was washed away during the synthesis process, resulting in non-specific binding sites. Therefore, the 1:4 ratio was retained as the optimal condition for subsequent experiments.

### 4.3. Adsorption Kinetics

Investigation of the adsorption kinetics of magnetic 3D COF–GSH MIPs or NIPs for the BSA target protein was conducted in PBS (pH = 6) containing 1 mg mL^−1^ of BSA. As presented in Figure 3d, the adsorption of BSA increased rapidly over the first 80 min, which was because of the imprinting sites present on the surface of the synthesized material. The adsorption reached equilibrium after 90 min, which is a much shorter rebinding time than that for some previously reported magnetic MIPs when considering BSA [31,32,33]. The kinetic rebinding of BSA from the magnetic 3D COF–GSH NIPs was much slower than that from the magnetic 3D COF–GSH MIPs because of nonspecific adsorption. These results suggest that surface imprinting technology can encourage distribution of imprinted sites on the external surface of magnetic 3D COF–GSH MIPs.

### 4.4. Isothermal Adsorption Properties

**Figure 4:**
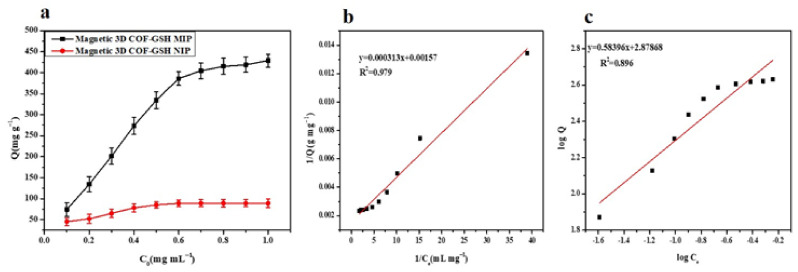
(**a**) Curves of adsorption isotherms of magnetic 3D COF–GSH MIPs and NIPs. (**b**) Adsorption isotherm equations of magnetic 3D COF–GSH MIPs Langmuir, and Freundlich (**c**).

As illustrated in Figure 4a, the amount of BSA adsorbed on the magnetic 3D COF–GSH MIPs and NIPs gradually increased as the initial concentration was increased; equilibrium was achieved for approximately 0.7 mg mL^−1^. The capacity of the magnetic 3D COF–GSH MIPs to adsorb BSA was considerably higher than that of the NIPs. This could have been because specific binding sites were present on the surface of the magnetic 3D COF–GSH MIPs, but only non-specific binding sites were present on NIPs. The binding capacity of the magnetic 3D COF–GSH MIPs was 429 mg g^−1^ and resulted in an IF of 4.79 when compared with the NIPs (89 mg g^−1^); this IF is much higher than in some previous studies, as listed in Table 1. This indicates that the magnetic 3D COF–GSH MIPs had a stronger ability to recognize the template BSA than the NIPs.

Figure 4b,c presents the adsorption isotherm data fitted to the Langmuir and Freundlich adsorption isotherm models, respectively. The Langmuir isotherm assumes that adsorption behavior is based on monolayer adsorption; thus, each recognition site adsorbs only one target molecule, the adsorption energy is equal at each recognition site, and no interactions occur between target molecules. The Freundlich model is a multilayer adsorption model and assumes that adsorption occurs on a heterogeneous surface and that affinity and adsorption energy of the adsorption materials for the target molecules are determined by specific surface properties [34]. In this study, the Langmuir adsorption isotherm (R^2^ = 0.979) model for the magnetic 3D COF–GSH MIPs was a better fit than that of the Freundlich adsorption isotherm (R^2^ = 0.896), which suggests that the adsorption process was a monolayer process.

### 4.5. Rebinding Selectivity

**Figure 5:**
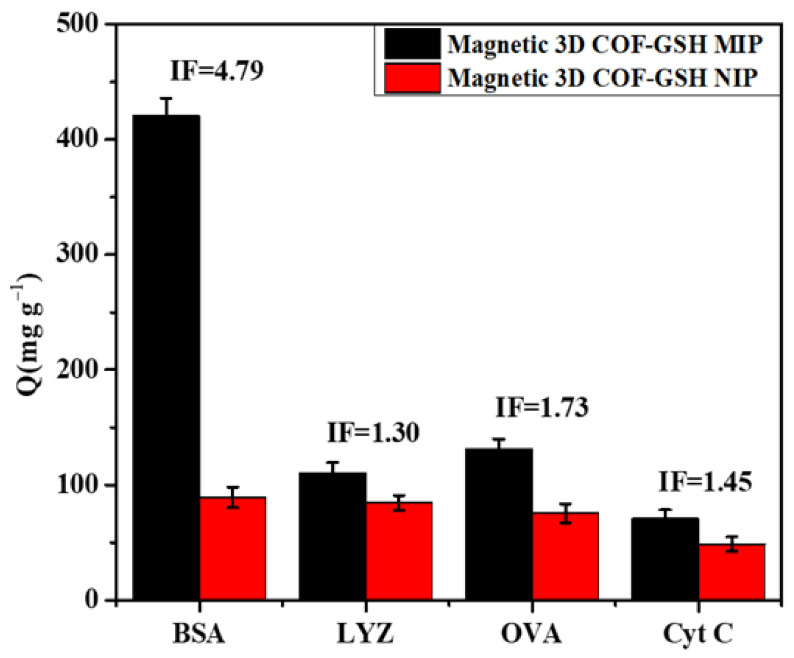
Rebinding selectivity of magnetic 3D COF–GSH MIPs and NIPs for different proteins.

OVA, Lyz, and Cyt C were used to investigate the BSA rebinding selectivity of the synthesized magnetic 3D COF–GSH MIPs. These proteins have different isoelectric points (pI) and molecular weights (Mw). OVA, Lyz, and Cyt C are smaller than BSA; the pIs of Lyz and Cyt C are 11.2 and 10.2 (>7), respectively, whereas the pI of OVA is 4.7 (<7) [34]. Figure 5 presents the rebinding selectivity of the magnetic 3D COF–GSH MIPs and NIPs for different proteins and the adsorption capacity results. The capacities of the magnetic 3D COF–GSH NIPs to adsorb the various proteins were not substantially different because of the absence of selective recognition sites. The magnetic 3D COF–GSH MIPs exhibited much higher selectivity for BSA than for the other proteins. The IF of the magnetic 3D COF–GSH MIPs for BSA, OVA, Lyz, and Cyt C were 4.79, 1.3, 1.73, and 1.45, respectively. The results obtained using Equation (3) indicated that the separation factors for Lyz, OVA, and Cyt C are 3.68, 2.76 and 3.30, respectively. The results indicated that the magnetic 3D COF–GSH MIPs have excellent specific recognition for BSA. The selectivity for BSA may be because the material’s specific recognition sites match the template BSA in terms of size, shape, and placement of functional groups.

### 4.6. Preliminary and Practical Application

**Figure 6:**
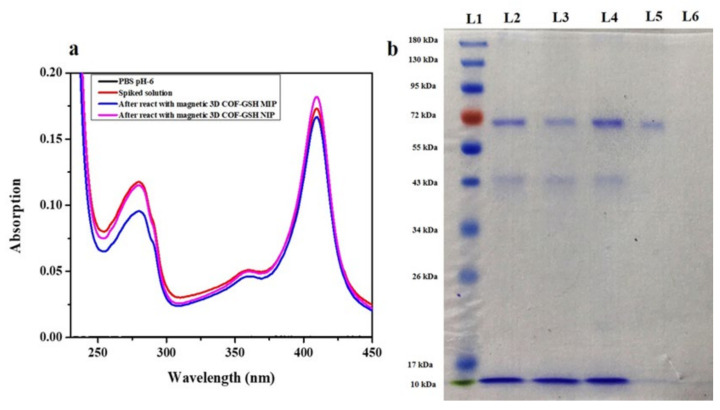
(**a**) UV–Vis analysis and (**b**) SDS–PAGE analysis for compatibility of magnetic 3D COF–GSH MIPs/NIPs. L1: Marker, L2: PBS with spiked proteins, L3; and L4: supernatant solutions after adsorption with magnetic 3D COF-GSH MIPs and NIPs respectively, L5; and L6: Eluted solutions from magnetic 3D COF-GSH MIPs and NIPs in 0.5% SDS and 0.1% HOAc (1:1) respectively.

The preliminary applications of the magnetic 3D COF–GSH MIP nanoparticles were investigated using a UV–vis spectrophotometry combined with SDS–PAGE gel electrophoresis analysis. PBS was spiked with known concentrations of the mixture of proteins and subjected to adsorption reactions with magnetic 3D COF–GSH MIPs and NIPs. Figure 6a presents the UV–Vis analysis. The absorbance of the supernatant solution at 280 nm after adsorption reaction with the magnetic 3D COF–GSH MIPs decreased more than when the magnetic 3D COF–GSH NIPs were employed. Similar results were also obtained in the SDS–PAGE analysis (Figure 6b). The upper lines of BSA and lower line of OVA were shown in L2–L4. The intensity of the corresponding BSA band in L3 was lower than that in L4, and the eluted solution L5 exhibited one less-intense band, corresponding to the point at which BSA separated from the complex solution. These results indicated that the magnetic 3D COF–GSH MIPs recognized BSA, but the process of BSA adsorption by the magnetic 3D COF–GSH NIPs remained nonspecific (L6). We also evaluated the practical applicability of the magnetic 3D COF–GSH MIP and NIP nanoparticles in FBS by using a UV–vis spectrophotometer, as illustrated in Figure S3. The absorbance of the supernatant solution at 280 nm after adsorption reaction with the magnetic 3D COF–GSH MIPs was decreased substantially more than that for the magnetic 3D COF–GSH NIPs, for which other absorption peaks had constant intensity. These results may have been caused by the differences in isoelectric point, molecular weight, and functional group distribution. BSA-imprinted sites exhibited a nonspecific affinity for other reference proteins. Similar results were also obtained in the SDS–PAGE analysis (Figure S3b). The intensity of the corresponding BSA band in L3 was lower than that in L4, and the eluted solution L5 exhibited one less-intense band, corresponding to the point at which BSA separated from the complex solution.

### 4.7. Regeneration Ability

Reusability of a material extends its practical applicability. Therefore, we performed five adsorption–desorption cycles with the magnetic 3D COF–GSH MIPs and NIPs to evaluate their regeneration ability. The BSA binding capacity of the magnetic 3D COF–GSH MIPs was 94% of the initial adsorption capacity after the five cycles. The decrease from 100% was because of incomplete elution of BSA and a few recognition sites being destructed during the repeated washing. However, the adsorption procedure of the magnetic 3D COF–GSH NIPs was nonspecific. Thus, the effect of the elution process could be disregarded, and no observable difference in absorption capacity was discovered.

## 5. Conclusions

This study proposed a simple and fast synthesis of magnetic 3D COFs through ultrasonication. Magnetic 3D COFs were functionalized with glutathione and combined with MIPs to form magnetic 3D COF–GSH MIP nanoparticles for separation and purification. The magnetic 3D COF–GSH MIP nanoparticles were characterized by their porosity (≈1 nm), surface functional groups, magnetization (32 emu g^−1^), and stability (94% after five adsorption–desorption cycles) for the solid-phase separation method. The synthesized materials from the proposed method showed high specific binding capacity (429 mg g^−1^) and a short equilibrium absorption time (1.5 h) for BSA imprinting. The proposed method has high imprinting (4.76) and selectivity (3.68-Lyz, 2.76-OVA, and 3.3-Cyt C) factors compared with those reported from recent studies on magnetic MIPs for BSA. Our study provides a new method of functional nanoparticles for the specific recognition of proteins and could be applied to the separation of complex biological samples.

## Figures and Tables

**Table 1:** Literature comparison of BSA imprinting on magnetic molecularly imprinted polymers (MMIP).

**Sample**	**Q_max__** **mg g^−1^**	**Linear Range_** **mg mL^−1^**	**IF**	**Equilibrium Absorption Time** **in Hour**	**Year and Reference**
MHNTs@PEG@4-VP MMIP	258	0.2–1.4	2.44	1.3	2020, [34]
HMS@MIPs.	431	0.0–0.8	2.01	2	2019, [30]
Fe_3_O_4_@void@BSA-pIL	130	0.1–0.5	2.79	1	2018, [35]
γ-Fe_2_O_3_@PIP	300	0.0–2.4	4	24	2017, [32]
Fe_3_O_4_@IL@MIP	51	0.1–0.9	3.33	1	2017, [36]
magnetic 3D COF–GSH MIPs	429	0.1–1.0	4.79	1.5	This work

Note: MHNTs@PEG@4-VP MMIP: PEG-coated, poly (4-vinylpyridine) modified magnetic halloysite nanotubes; HMS@MIPs: Hollow Fe_3_O_4_@SiO_2_@BSA-molecular imprinted polymers; Fe_3_O_4_@void@BSA–pIL: magnetic hollow molecular imprinted poly (ionic liquids) nanospheres; γ–Fe_2_O_3_@PIP: γ–Fe_2_O_3_ protein imprinted polymers; Fe_3_O_4_@IL@MIP: Ionic liquid functionalized Fe_3_O_4_ nanoparticle molecular imprinted polymers.

## Data Availability

Not applicable.

## Supplementary Materials

The following supporting information can be downloaded at: https://www.mdpi.com/article/10.3390/nano12030411/s1, Scheme S1: Synthesis of cTp. Figure S1: FT-IR spectra of Tp and cTp (a) and enlarged(b). Figure S2: N2 adsorption-desorption isotherm and pore size distribution(inset) of magnetic 3D COFs. Figure S3: XRD of magnetic 3D COF nanoparticles. Figure S4: (a) UV-vis spectrum and (b) SDS-PAGE of practical analysis of magnetic 3D COF-GSH MIPs. Table S1: Optimization of amounts of TEOS and APTES in synthesis reaction of magnetic 3D COF-GSH MIPs/NIPs.

## References

[1] Côté A.P., Benin A., Ockwig N., Okeeffe M., Matzger A., Yaghi O. (2005). Covalent Organic Frameworks. Science.

[2] Liu L.H., Yang C.X., Yan X.P. (2017). Methacrylate-bonded covalent-organic framework monolithic columns for high performance liquid chromatography. J. Chromatogr. A.

[3] Ding S.Y., Wang W. (2013). Covalent organic frameworks (COFs): From design to applications. Chem. Soc. Rev..

[4] Zhang Y., Riduan S.N. (2012). Functional porous organic polymers for heterogeneous catalysis. Chem. Soc. Rev..

[5] Gao Q., Li X., Ning G., Leng K., Tian B., Liu C., Tang W., Xu H., Loh K. (2018). Highly photoluminescent two-dimensional imine-based covalent organic frameworks for chemical sensing. Chem Commun.

[6] Hynek J., Zelenka J., Ruthousky J., Kubat P., Ruml T., Demel J., Lang K. (2018). Designing Porphyrinic Covalent Organic Frameworks for the Photodynamic Inactivation of Bacteria. ACS Appl. Mater. Interfaces.

[7] Lohse M.S., Bein T. (2018). Covalent organic frameworks: Structures, synthesis, and applications. Adv. Funct. Mater..

[8] Yan X., Song Y., Liu J., Zhou N., Zhang C., He L., Zhang Z., Liu Z. (2019). Two-dimensional porphyrin-based covalent organic framework: A novel platform for sensitive epidermal growth factor receptor and living cancer cell detection. Biosens. Bioelectron..

[9] El-Kaderi H.M., Hunt J.R., Mendoza-Cortes J., Corte A., Taylor R., Okeeffe M., Yaghi O. (2007). Designed Synthesis of 3D Covalent Organic Frameworks. Science.

[10] Díaz U., Corma A. (2016). Ordered covalent organic frameworks, COFs and PAFs. From preparation to application. Coord. Chem. Rev..

[11] Xiang Z., Dai Q., Chen J., Dai L. (2016). Edge functionalization of graphene and two-dimensional covalent organic polymers for energy conversion and storage. Adv. Mater..

[12] Yuan S., Li X., Zhu J., Zhang G., Puyvelde P., Bruggen B. (2019). Covalent organic frameworks for membrane separation. Chem. Soc. Rev..

[13] Li N., Wu D., Hu N., Fang G., Li X., Sun J., Chen X., Suo Y., Li G., Wu Y. (2018). Effective Enrichment and Detection of Trace Polycyclic Aromatic Hydrocarbons in Food Samples based on Magnetic Covalent Organic Framework Hybrid Microspheres. J. Agric. Food Chem..

[14] Yan Y., Lu Y., Wang B., Gao Y., Zhao L., Liang H., Wu D. (2018). Self-assembling hydrophilic magnetic covalent organic framework nanospheres as a novel matrix for phthalate ester recognition. ACS Appl. Mater. Interfaces.

[15] Wang Q., Wu H., Lv F., Cao Y., Zhou Y., Gan N. (2018). A headspace sorptive extraction method with magnetic mesoporous titanium dioxide@covalent organic frameworks composite coating for selective determination of trace polychlorinated biphenyls in soils. J. Chromatogr. A.

[16] Luo B., Yan S., Zhang Y., Zhou J., Lan F., Wu Y. (2021). Bifunctional magnetic covalent organic framework for simultaneous enrichment of phosphopeptides and glycopeptides. Anal. Chim. Acta.

[17] Guan X., Chen F., Fang Q., Qiu S. (2020). Design and applications of three dimensional covalent organic frameworks. Chem. Soc. Rev..

[18] Zhao Q., Zhang H., Zhao H., Liu J., Liu J., Chen Z., Li B., Liao X. (2020). Strategy of Fusion Covalent Organic Frameworks and Molecularly Imprinted Polymers: A Surprising Effect in Recognition and Loading of Cyanidin-3-O-glucoside. ACS Appl. Mater. Interfaces.

[19] Jahanban-Esfahlan A., Roufegarinejad L., Jahanban-Esfahlan R., Tabibiazar M., Amarowicz R. (2020). Latest developments in the detection and separation of bovine serum albumin using molecularly imprinted polymers. Talanta.

[20] Zhao Q.Y., Zhao H., Yang X., Zhang H., Dong A., Wang J., Li B. (2018). Selective recognition and fast enrichment of anthocyanins by dummy molecularly imprinted magnetic nanoparticles. J. Chromatogr. A.

[21] Zhao Q., Ma C., Liu J., Chen Z., Zhao H., Li B., Yang X. (2021). Synthesis of magnetic covalent organic framework molecularly imprinted polymers at room temperature: A novel imprinted strategy for thermo-sensitive substance. Talanta.

[22] Zhou R., Shen N., Zhao J., Su Y., Ren H. (2018). Glutathione-coated Fe_3_O_4_ nanoparticles with enhanced Fenton-like activity at neutral pH for degrading 2,4-dichlorophenol. J. Mater. Chem. A.

[23] Vinluan R.D., Liu J., Zhou C., Yu M., Yang S., Kumar A., Sun S., Dean A., Sun X., Zheng J. (2014). Glutathione-coated luminescent gold nanoparticles: A surface ligand for minimizing serum protein adsorption. ACS Appl. Mater. Interfaces.

[24] Luo B., He J., Li Z., Lan F., Wu Y. (2019). Glutathione-Functionalized Magnetic Covalent Organic Framework Microspheres with Size Exclusion for Endogenous Glycopeptide Recognition in Human Saliva. ACS Appl. Mater. Interfaces.

[25] Matsumoto T., Chiba J. (1990). Rheological and small-angle X-ray scattering investigations on the shape and ordered arrangement of native ovalbumin molecules in aqueous colloids. J. Chem. Soc. Faraday Trans..

[26] Yuan S., Deng Q., Fang G., Wu J., Li W., Wang S. (2014). Protein imprinted ionic liquid polymer on the surface of multiwall carbon nanotubes with high binding capacity for lysozyme. J. Chromatogr. B.

[27] Tsai H.Y., Wu H.H., Chou B.C., Li C.S., Gau B.Z., Lin Z.Y., Fuh C.B. (2019). A magneto-microfluidic platform for fluorescence immunosensing using quantum dot nanoparticles. Nanotechnology.

[28] Lu F., Lin J., Lin C., Qi G., Lin X., Xie Z. (2021). Heteroporous 3D covalent organic framework-based magnetic nanospheres for sensitive detection of bisphenol A. Talanta.

[29] Luo B., Li G., Li Z., He J., Zhou J., Wu L., Lan F., Wu Y. (2021). Construction of a magnetic covalent organic framework with synergistic affinity strategy for enhanced glycopeptide enrichment. J. Mater. Chem. B.

[30] Liu Z., Wang Y., Xu F., Wei X., Chen J., Li H., He X., Zhou Y. (2020). A new magnetic molecularly imprinted polymer based on deep eutectic solvents as functional monomer and cross-linker for specific recognition of bovine hemoglobin. Anal. Chim. Acta.

[31] Zhou J., Wang Y., Ma Y., Zhang B., Zhang Q. (2019). Surface molecularly imprinted thermo-sensitive polymers based on light-weight hollow magnetic microspheres for specific recognition of BSA. Appl. Surf. Sci..

[32] Boitard C., Rollet A., Menager C., Griffete N. (2017). Surface-initiated synthesis of bulk-imprinted magnetic polymers for protein recognition. Chem Commun.

[33] Gai Q.-Q., Qu F., Zhang T., Zhang Y. (2011). The preparation of bovine serum albumin surface-imprinted superparamagnetic polymer with the assistance of basic functional monomer and its application for protein separation. J. Chromatogr. A.

[34] Li X., Liu H., Deng Z., Chen W., Li T., Zhang Y., Zhang Z., He Y., Tan Z., Zhong S. (2020). Pegylated thermo-sensitive bionic magnetic core-shell structure molecularly imprinted polymers based on halloysite nanotubes for specific adsorption and separation of bovine serum albumin. Polymers.

[35] Fan J.P., Yu J., Yang X., Zhang X., Yuan T., Peng H. (2018). Preparation, characterization, and application of multiple stimuli-responsive rattle-type magnetic hollow molecular imprinted poly (ionic liquids) nanospheres (Fe_3_O_4_@void@PILMIP) for specific recognition of protein. Chem. Eng. J..

[36] Qian L., Sun J., Hou C., Yang J., Li Y., Lei D., Yang M., Zhang S. (2017). Immobilization of BSA on ionic liquid functionalized magnetic Fe_3_O_4_ nanoparticles for use in surface imprinting strategy. Talanta.

