# Supplementary material for: Functional Nanoparticles with Magnetic 3D Covalent Organic Framework for the Specific Recognition and Separation of Bovine Serum Albumin
Source: Nanomaterials (Basel). 2022 Jan 26;12(3):411. doi: 10.3390/nano12030411 (PMC8840370; doi:10.3390/nano12030411)
Supplement: Supplementary file 1 [file nanomaterials-12-00411-s001.zip › nanomaterials-1554054-supplementarym1.pdf]

Supplementary Materials

Functional Nanoparticles with Magnetic 3D Covalent Organic Framework for the Specific Recognition and Separation of Bovine Serum Albumin

Lokesh Bettada [1], Hweiyan Tsai [2,3,*] and C. Bor Fuh [1,*]

[1] Department of Applied Chemistry, National Chi Nan University, Nantou 545, Taiwan; s105324905@mail1.ncnu.edu.tw
[2] Department of Medical Applied Chemistry, Chung Shan Medical University, Taichung 402, Taiwan
[3] Department of Medical Education, Chung Shan Medical University Hospital, Taichung 402, Taiwan
* Correspondence: annetsai@csmu.edu.tw (H.T.); cbfuh@ncnu.edu.tw (C.B.F.); Tel.: +886-49-2919-779 (C.B.F.)

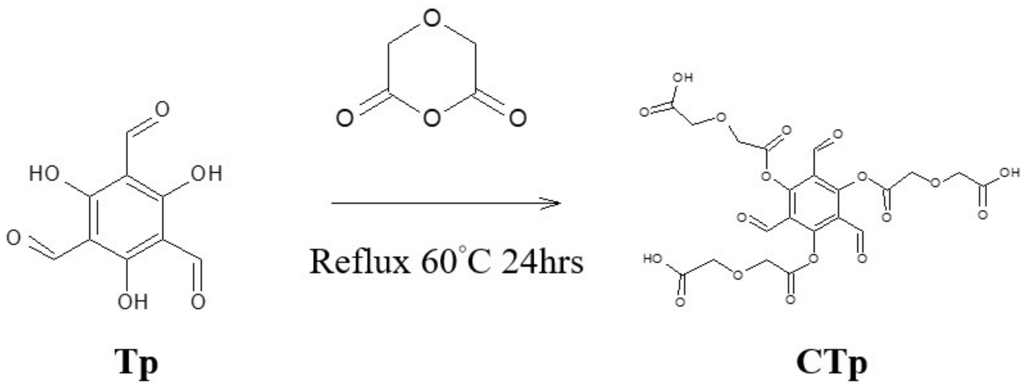

Scheme S1. Synthesis of cTp.

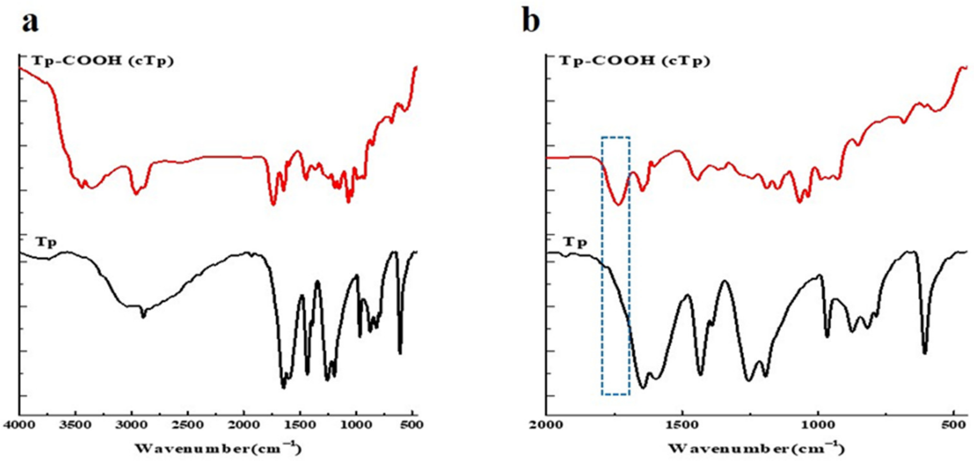

Figure S1. FT-IR spectra of Tp and cTp (a), enlarged(b).

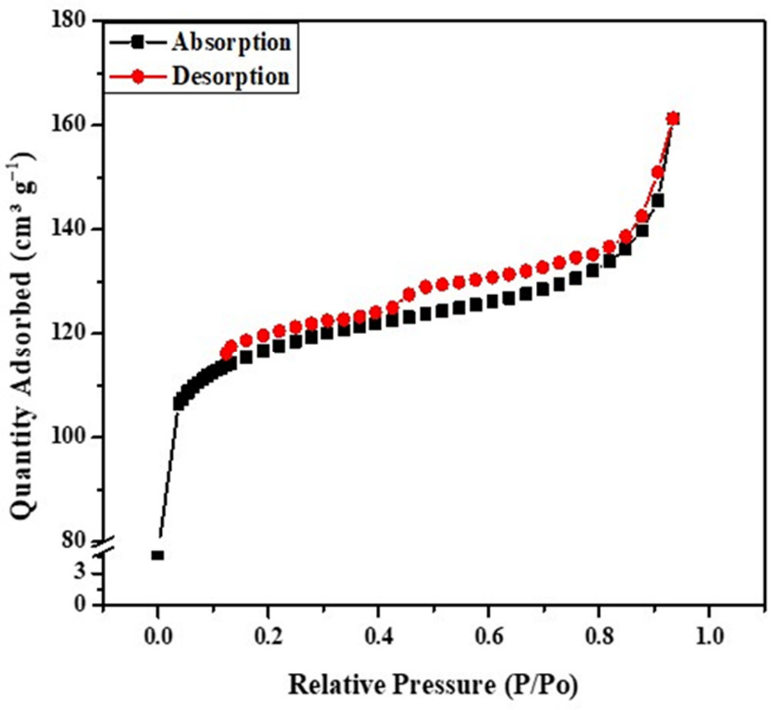

Figure S2. N_2 adsorption-desorption isotherm and pore size distribution(inset) of magnetic 3D COFs.

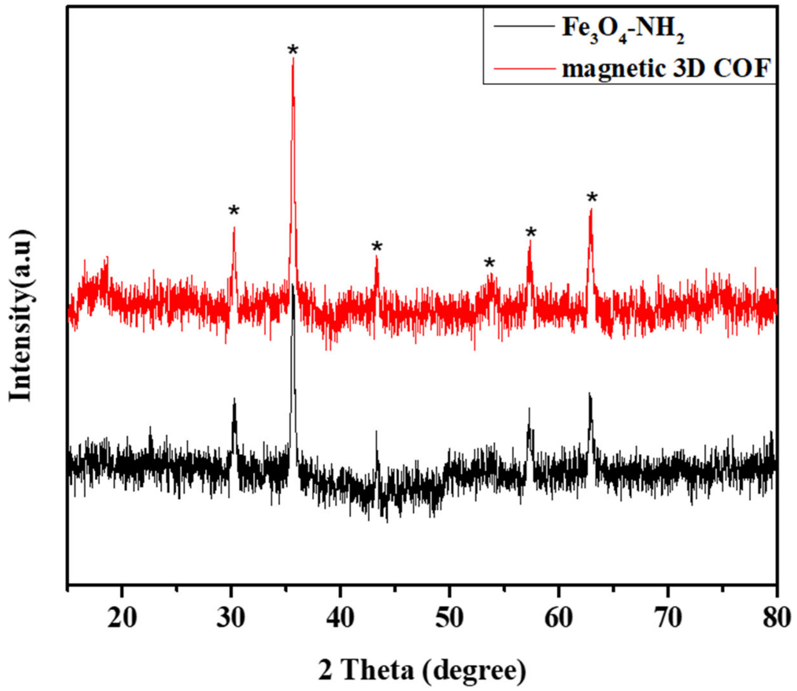

Figure S3. XRD of magnetic 3D COF nanoparticles. Two θ values and crystal planes 30.2° (220), 35.4° (311), 43.2° (400), 53.4° (422), 57.2° (511), and 62.4° (440) were characteristic peaks of Fe_3O_4.

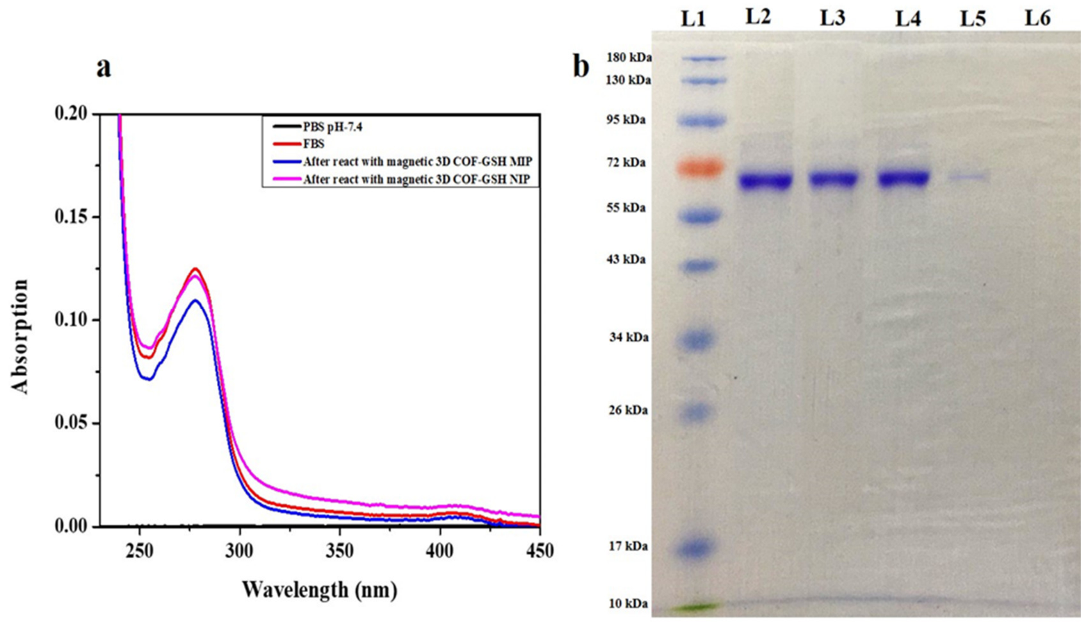

Figure S4. (a) UV-vis spectrum and (b) SDS-PAGE of practical analysis for fetal bovine serum of magnetic 3D COF-GSH MIPs. L1: Marker, L2: Fetal bovine serum (FBS), L3; and L4: supernatant solution after adsorption with magnetic 3D COF-GSH MIPs and NIPs respectively, L5; and L6: Eluted solution from magnetic 3D COF-GSH MIPs and NIPs in 0.5% SDS and 0.1 % HOAc (1:1) respectively.

Table S1. Optimization of amounts of TEOS and APTES in synthesis reaction of magnetic 3D COF-GSH MIPs/NIPs.

Amount of Magnetic 3D COF-GSH(mg)	Amount of TEOS(μL)	Amount of APTES(μL)	Q $_{\text{magnetic 3D COF MIPs}}$ (mg g^{-1})	Q $_{\text{magnetic 3D COF NIPs}}$ (mg g^{-1})
3	50	10	201.2±15.1	62.5±5.2
		20	352.2±10.3	87.2±9.5
		30	251.3±11.3	74.2±8.6
	10	30	281.3±9.6	68.3±8.6
	30		248.3±12.5	59.5±7.8
	60		208.6±14.6	56.5±6.6
